# Noise Stress Abrogates Structure-Specific Endonucleases within the Mammalian Inner Ear

**DOI:** 10.3390/ijms25031749

**Published:** 2024-02-01

**Authors:** O’neil W. Guthrie

**Affiliations:** Cell & Molecular Pathology Laboratory, Department of Communication Sciences and Disorders, Northern Arizona University, Flagstaff, AZ 86011, USA; oneil.guthrie@nau.edu; Tel.: +1-928-523-8142

**Keywords:** noise stress, treatment, sensorineural, cochlea, endonuclease

## Abstract

Nucleotide excision repair (NER) is a multistep biochemical process that maintains the integrity of the genome. Unlike other mechanisms that maintain genomic integrity, NER is distinguished by two irreversible nucleolytic events that are executed by the xeroderma pigmentosum group G (XPG) and xeroderma pigmentosum group F (XPF) structure-specific endonucleases. Beyond nucleolysis, XPG and XPF regulate the overall efficiency of NER through various protein–protein interactions. The current experiments evaluated whether an environmental stressor could negatively affect the expression of *Xpg (Ercc5: excision repair cross-complementing 5)* or *Xpf (Ercc4: excision repair cross-complementing 4)* in the mammalian cochlea. Ubiquitous background noise was used as an environmental stressor. Gene expression levels for *Xpg* and *Xpf* were quantified from the cochlear neurosensory epithelium after noise exposure. Further, nonlinear cochlear signal processing was investigated as a functional consequence of changes in endonuclease expression levels. Exposure to stressful background noise abrogated the expression of both *Xpg* and *Xpf*, and these effects were associated with pathological nonlinear signal processing from receptor cells within the mammalian inner ear. Given that exposure to environmental sounds (noise, music, etc.) is ubiquitous in daily life, sound-induced limitations to structure-specific endonucleases might represent an overlooked genomic threat.

## 1. Introduction

Arguably, one of the most important molecular mechanisms within the cells of humans and other placental mammals is the nucleotide excision repair (NER) mechanism. This multistep biochemical process is responsible for maintaining the integrity of the genome. It localizes and removes a large variety of structural and chemical alterations to DNA. For instance, the molecules that complement the NER process are sensitive to small DNA damage products caused by endogenous metabolic by-products as well as large-bulky helix distorting cross-links caused by exogenous exposures [1,2]. Therefore, NER protects the integrity of both active and inactive genes under normal conditions and during episodes of environmental stress. By protecting genes and genomes, NER can maintain the stability of cellular physiology. It is known that human mutations to NER genes result in degeneration of cells within the inner ear, which leads to permanent and severe hearing loss [3]. This suggests that NER is necessary for protecting inner ear genetic material from endogenous metabolic by-products. A series of experiments localized and quantified the expression of various NER factors within the mammalian inner ear [4,5,6]. Then, challenge studies that employed both chemical and physical stressors concluded that NER is a general stress response that is deployed by the inner ear for protection [7,8,9].

**Figure 1:**
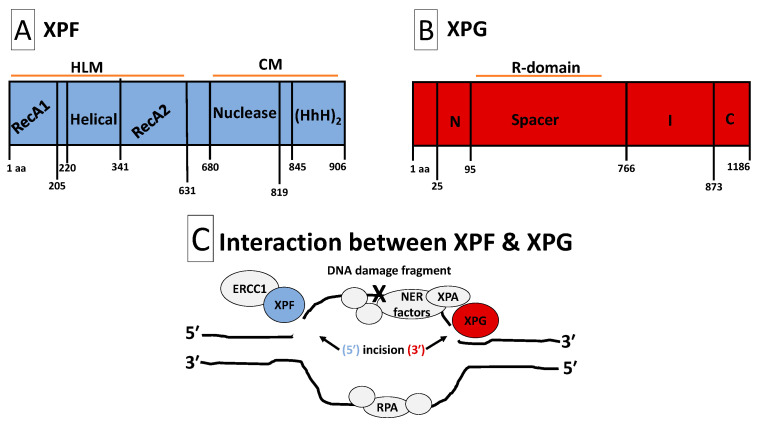
Structures and interaction of XPF and XPG. Panel (**A**): Structure of XPF. XPF is composed of HLM and CM domains. The HLM domain is related to the superfamily-2-helicases and is required for ds/ssDNA binding, protein–protein interactions, and full XPF activity. It is constituted with two RecA-like subdomains (RecA1 and RecA2) that flank an all α-helical subdomain. The CM domain facilitates heterodimerization with ERCC1 to stabilize XPF, perpetuate protein–protein interactions and form functional units. Additionally, the CM contains a metal-mediated V/IERKX_3_D nuclease motif and a tandem (HhH)_2_ motif. Panel (**B**): Structure of XPG. XPG is composed of two conserved nuclease domains, called N-region and I-region. The N- and I-regions (the nuclease core) are separated by an acidic spacer region (called R-domain) that is unique to XPG. XPG also contains a PIP-box motif that facilitates interaction with PCNA, ubiquitin-binding motifs and three nuclear localization signals. The C-terminus of XPG contains a disordered region of coiled coils. Such disordered coiled coils are also found in the spacer region. Protein–protein interactions with several TFIIH subunits (XPB, XPD, p52, p44 and p62) and RPA will occur at the spacer region and C- and N-termini. Panel (**C**): Interaction between XPF and XPG. NER factors unwind the DNA around the DNA damage, while RPA safeguards the undamaged strand. XPA and RPA position the ERCC1-XPF heterodimer for 5′ endonuclease activity, followed by XPG 3′ endonuclease activity that flanks the DNA damage fragment. After removal of the damaged DNA strand, the gap is re-synthesized with the help of DNA repair factors including PCNA and DNA polymerases. Abbreviations: aa, amino acid; HLM, helicase-like module; CM, catalytic module; ds/ssDNA, double-/single-stranded DNA junction; RecA1/2, recombinase A1/2; ERCC1, excision repair cross-complementation group 1; HhH_2_, double helix-hairpin-helix motif; R-domain, coiled coils spacer region; C, extended coiled coils C-terminus; PIP-box, PCNA-interacting protein-box; NER, nucleotide excision repair; PCNA, proliferating cell nuclear antigen; RPA, replication protein-A; XPA/B/D/F/G, xeroderma pigmentosum group A/B/D/F/G.

Relative to other mammalian DNA repair mechanisms, NER is distinguished by two nucleolytic events that excise a stretch of oligonucleotides that contain damaged DNA [10,11]. The two nucleolytic events represent the first irreversible step within the NER reaction and are accomplished by two structure-specific endonucleases called xeroderma pigmentosum group G (XPG) and xeroderma pigmentosum group F (XPF) [12]. Figure 1 illustrates the structures and interactions of XPG and XPF. The XPG endonuclease contains an N-terminal nuclease domain (N-nuclease) and a more internal nuclease domain (I-nuclease) [13]. Together, the N- and I-nuclease domains form the nucleolytic core of the molecule [13,14]. A highly acid 600 amino acid spacer region separates the two nucleolytic domains [15]. This spacer region, which has no known structural motifs, mediates several protein–protein interactions that are critical to NER. For instance, the highly disordered spacer region mediates XPG’s interaction with XPB, XPD and RPA [12,16,17,18]. The C-terminal region of XPG participates in protein–protein interactions with multiple TFIIH subunits such as p62, p44, XPB and XPD [16,18]. Contained within the XPG molecule is a PCNA interacting domain that anchors and allows PCNA to participate in perpetuating the terminal steps of NER [19,20]. XPB, XPD, RPA, p62, p44 and PCNA are all important NER factors [2]. Therefore, beyond incision, it appears that the XPG endonuclease is also important for maintaining protein–protein interactions that are necessary for NER [21]. A similar conclusion can be reached for XPF. For instance, the C-terminal of XPF contains two helix-harpin-helix motifs that facilitate dimerization with ERCC1 [22,23,24,25]. Indeed, XPF forms a complex with ERCC1, and ERCC1 harbors a central domain that binds and stabilizes XPA [12]. Furthermore, there is some evidence of protein–protein interactions between XPF and RPA [26]. Both ERCC1 and XPA are critical players in NER [2]. Although, dual incision by XPG and XPF are distinguishing features of NER, mounting evidence has now indicated that these two endonucleases also possess structural roles via protein–protein interactions that are necessary for completing the NER process [12]. Therefore, an understanding of XPG and XPF expression is important for overall cellular physiology.

It is estimated that 600 million individuals worldwide are at risk for exposure to loud noise that may cause hearing loss, and such noise-induced hearing loss (NIHL) may account for ⅓ of all hearing loss in some countries [27,28]. In the USA, one in six adults report hearing difficulties, and 26 million have NIHL due to occupational and recreational activities [29]. The number of children with NIHL has significantly increased from a decade ago such that 16% of children have NIHL, and this is now expected to reach epidemic levels since 80–90% of children listen to personal listening devices (cell phones, iPod, etc.,) via earphones for a significant proportion of their day, and these devices have been shown to emit levels that are hazardous [30,31]. Interestingly, noise stress has been shown to induce various types of DNA damage products within the inner ear, which indicates that DNA damage may be an important part of the pathophysiology of hearing loss [9,32,33,34]. Noise stress also mobilizes NER within the inner ear as a defensive response, yet noise stress is consistently successful at perpetuating the accumulation of various types of DNA damage products, leading to disordered auditory functions [7,9,32]. This discrepancy is hard to rationalize, and it might seem that inner ear NER is not protective, since its mobilization during noise stress fails to preserve auditory functions. However, therapeutic mobilization of inner ear NER has been shown to be protective by preventing and even rescuing auditory functions after severe noise trauma [7]. Therefore, it is possible that the mobilization of NER due to noise stress is somehow corrupted. The current series of experiments investigated the effect of noise stress on inner ear expression of *Xpg (Ercc5: excision repair cross-complementing 5)* and *Xpf (Ercc4: excision repair cross-complementing 4)*. Given that XPG and XPF are important for regulating the overall efficiency of NER through protein–protein interactions as well as dual incision, it was posited that noise stress may fail to mobilize these two endonucleases, which would provide a basis to understand why noise-induced mobilization of NER is not protective. The principal findings revealed that exposure to noise abrogates the expression of both *Xpg* and *Xpf*, and these effects were associated with pathological nonlinear signal processing from receptor cells within the mammalian inner ear.

## 2. Results

### 2.1. Expression of Structure-Specific Endonucleases

**Figure 2:**
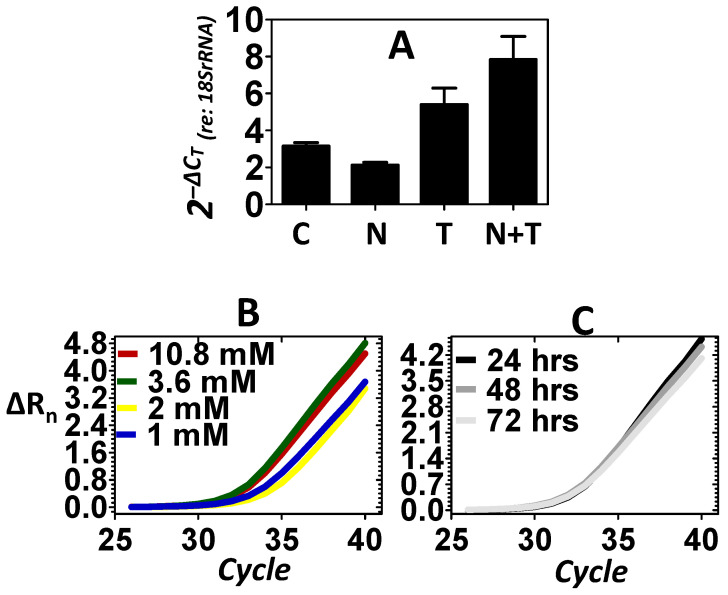
Expression of the *Xpg* endonuclease. Control (C), noise (N) stress, telomere (T) treatment and combined noise + telomere (N + T) exposure were used to evaluate the expression of *Xpg*. (**A**): Noise stress failed to induce a substantive increase in the expression of *Xpg*; however, telomere treatment elicited a significant increase in the expression of *Xpg*. Interestingly, the combination of noise stress with telomere treatment (noise + telomere) yielded the largest increase in *Xpg* expression—an indication that noise + telomere is an effective inducer of endonuclease expression. The ordinate is gene expression relative to the expression of the 18S rRNA (internal control). Each bar represents the mean ± S.E. (**B**,**C**): Telomere’s capacity for inducing *Xpg* expression was further confirmed in dose–response (Panel B) and time-course (Panel C) experiments. Note that *Xpg* expression increased as telomere concentration (1 to 10.8 mM) increased, and this increase in *Xpg* expression persisted over 3 days (72 h). Panels B and C are the measured emission spectra (ΔR_n_) as a function of the real-time polymerase reaction cycles.

Previous experiments have shown that under stressful conditions, the inner ear may respond with increased gene expression, leading to subcellular nuclear translocation of NER factors as a defensive reaction [8,9]. Exposure to loud noise is a potent and specific stressor to the cells of the inner ear. Therefore, experiments were conducted to evaluate whether exposure to loud noise would induce the expression of structure-specific endonucleases within the inner ear as a defense against noise stress. An 8 kHz octave band noise (OBN) @ 105 dB SPL was used in the current experiments. This particular noise dose has previously been shown to consistently induce DNA damage within the inner ear [32]. Figure 2A shows that relative to control, the noise stress failed to induce the expression of *Xpg*. Previous experiments have shown that telomere treatment can mobilize NER factors within the mammalian inner ear and this effect preserves cellular physiology under stressful conditions [7]. Therefore, telomere was directly delivered to the ear via transtympanic injection as a secondary method of inducing the expression of *Xpg*. Figure 2A shows changes in the expression of the *Xpg* endonuclease in response to the telomere treatment. Relative to control, telomere treatment resulted in an increase in the expression of *Xpg*. Figure 2A also reveals that telomere treatment after the noise exposure (noise + telomere) resulted in a further increase in the expression of *Xpg*. This suggested that noise + telomere was a more effective inducer of *Xpg* than either approach alone. Statistical analyses (Table 1) demonstrated that the expression levels of *Xpg* are dependent on treatment condition (main effect of treatment: *p* < 0.01). Furthermore, Dunnett’s pairwise comparison testing confirmed that the noise + telomere condition (Dunnett: *p* < 0.01) resulted in statistically significant expression of *Xpg* relative to control. However, neither noise exposure alone nor telomere treatment alone (Dunnett: *p* > 0.05) could statistically increase expression of *Xpg* relative to control. Both dose–response and time-course experiments were then pursued to further verify that the noise + telomere condition could induce *Xpg* expression. Figure 2B reveals that a systematic change in the concentration of telomere from 1 to 10.8 mM resulted in a systematic increase in the expression level of *Xpg*. Figure 2C further reveals that the noise + telomere induction of *Xpg* was stable out to 3 days after induction (telomere concentration in Figure 2C is 3.6 mM).

**Figure 3:**
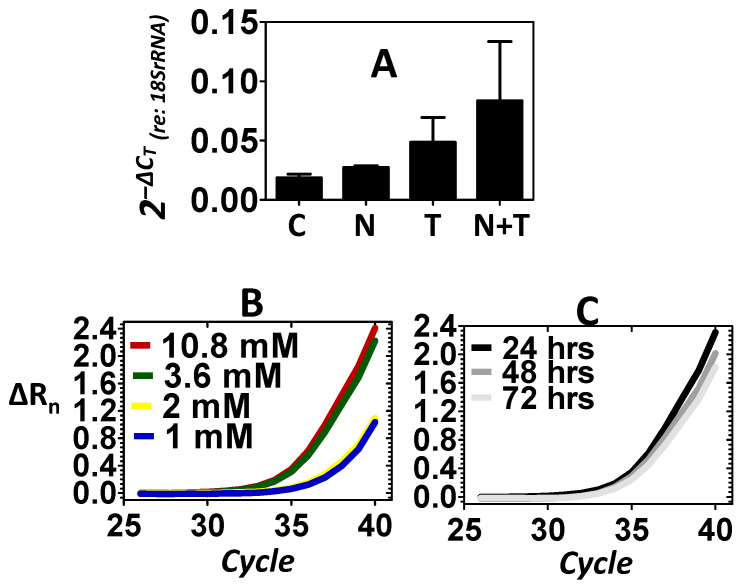
Expression of the *Xpf* endonuclease. Control (C), noise (N) stress, telomere (T) treatment and combined noise+telomere (N+T) exposure were used to evaluate the expression of *Xpf*. Panel (**A**): Noise stress failed to induce a significant increase in the expression of *Xpf*; however, telomere treatment induced a noticeable increase in the expression of *Xpf*. The combination of noise stress with telomere treatment (noise + telomere) yielded the largest increase in *Xpf* expression—an indication that noise + telomere is an effective inducer of endonuclease expression. The ordinate is gene expression relative to the expression of the 18S rRNA (internal control). Each bar represents the mean ± S.E. Panels (**B**,**C**): Telomere’s capacity for inducing *Xpf* expression was further confirmed in dose–response (Panel B) and time-course (Panel C) experiments. Note that *Xpf* expression increased as telomere concentration (1 to 10.8 mM) increased, and this increase in *Xpf* expression persisted over 3 days (72 h). Panels B–C are the measured emission spectra (ΔR_n_) as a function of the real-time polymerase reaction cycles.

In addition to *Xpg*, the expression of the *Xpf* endonuclease was also evaluated. Figure 3A shows that relative to control, the noise did not induce the expression of *Xpf*. However, relative to control, telomere treatment resulted in an increase in the expression of *Xpf*, which provided another example that telomere may perpetuate the mobilization of structure-specific endonucleases. Figure 3A also reveals that combining noise stress with telomere treatment (noise + telomere) resulted in the greatest increase in the expression of *Xpf*. This suggested that noise + telomere was a more effective inducer of *Xpf* than the noise-only or telomere-only conditions. To further confirm the effects of telomere on the expression of *Xpf*, both dose–response and time-course experiments were conducted. Figure 3B shows that the level of *Xpf* could be increased as the concentration of telomere increased. Figure 3C further reveals that telomere’s induction of *Xpf* was somewhat stable out to 3 days after induction (telomere concentration in Figure 3C is 3.6 mM). Statistical analyses (Table 1) on the expression levels of *Xpf* did not support an effect of the treatment condition (main effect of treatment: *p* > 0.05), therefore, the biological significance of the noise exposure’s failure to induce endonuclease expression was evaluated relative to telomere’s successful induction of endonucleases within the inner ear.

### 2.2. Biological Significance of Endonuclease Expression

The inner ear is an exquisite signal detector that processes frequency-specific vibrations that range from 0.1 nm, the diameter of a single hydrogen atom, to vibrations that are over a thousand billion times louder (e.g., an explosion) [35]. The cellular substrates that underlie such extensive processing are the receptor hair cells [36]. The inherent nonlinear signal processing of these receptor cells accounts for the inner ear’s remarkable range of signal detection. A diagnostic and functional feature of this nonlinear signal processing is the generation of distortion products [37]. Distortion products are generated as a result of nonlinear signaling processing and serve as a sensitive measure of the functional integrity of receptor cells [38]. To determine the biological significance of experimental manipulations of endonuclease expression within the inner ear; distortion products were recorded after noise exposure and after combined exposure to noise + telomere. If induction of endonuclease expression within the inner ear has a positive biological effect, then one would expect an associated improvement in nonlinear signal processing. In these experiments, both ears of the animals were exposed to hazardous noise. At the end of the noise exposure, only one ear received telomere. Therefore, each animal had one ear that was exposed to noise-only and one ear that was treated with noise, then telomere (noise + telomere). Seven and 60 days later, distortion products were recorded from both ears to study receptor nonlinearity.

**Figure 4:**
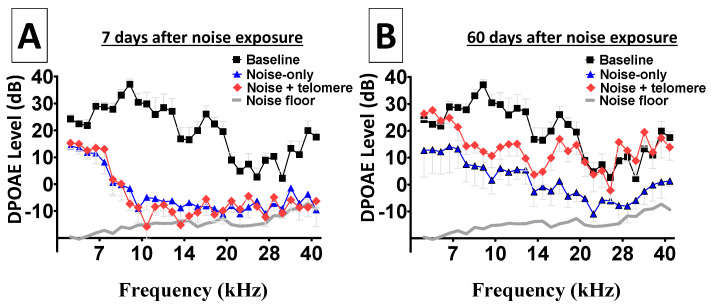
Improvement in nonlinear cochlear processing. Nonlinear signal processing by sound sensing receptor cells can be depleted after exposure to hazardous noise levels; therefore, the improvement in receptor-generated distortion product otoacoustic emissions (DPOAEs) allows further investigation of the physiological significance of endonuclease expression. Panel (**A**): Both ears from each animal were exposed to noise; then, immediately after the noise exposure, only one ear from each animal received telomere. At 7 days after the noise exposure, both the noise-exposure-only ears and the noise-plus-telomere-treated ears showed depleted DPOAEs—an indication that both ears suffered similar loss of nonlinear cochlear processing. Panel (**B**): At 60 days after the noise exposure, the noise-exposure-only ears exhibited modest improvements in DPOAEs. However, the noise-plus-telomere-treated ears showed improvements that approximated baseline levels in some frequency bands. Each data point is the mean ± S.E. The X-axis in both panels represents *F*_2_ frequencies.

Figure 4A reveals depleted distortion products from both ears, early (7 days) after noise exposure. This is particularly important because hazardous noise exposure is known to permanently abolish the nonlinear signal processing from receptor cells, leading to depleted distortion products. Therefore, Figure 4A suggests that both ears suffered similar loss of DPOAE activity. Figure 4B shows long-term (60 days) depletion of distortion products from both ears. Note that ears treated with only the hazardous noise continued to exhibit depleted DPOAE across the entire frequency range. Interestingly, there was a modest level of improvement in DPOAE levels from the noise-treated ears. However, ears that were treated with noise and then received telomere exhibited more dramatic improvements in DPOAE levels. In fact, the lower and higher frequency regions showed improved DPOAE levels that approximated levels recorded before the noise exposure (baseline). Such improvements in cochlear nonlinear processing (in the current experimental context) provided a unique opportunity to study the biological significance of experimental manipulations of endonuclease expression within the cochlea. Statistical computations further confirmed the improvement in DPOAE levels with telomere treatment relative to noise-only exposure. At 7 days after the noise exposure, a Bonferroni pairwise comparison showed that there was no statistically significant (*p >* 0.05) difference between DPOAE recordings from ears exposed to noise only relative to ears treated with noise + telomere. This is consistent with the fact that ears treated with noise and telomere suffered similar loss of cochlear signal processing (see Figure 4). However, at 60 days after the noise exposure, a Bonferroni pairwise comparison showed that there was a statistically significant (*p <* 0.01) difference between DPOAE recordings from ears exposed to noise only relative to ears treated with noise + telomere. As revealed in Figure 4, ears treated with noise + telomere exhibited more robust cochlear signal processing relative to ears treated with noise only.

## 3. Discussion

It is known that exposure to stressful noise can induce the accumulation of damaged DNA within the mammalian inner ear, and this effect is associated with pathological auditory functions and even dead inner ear cells [32]. Previous experiments have documented the existence of NER within the mammalian inner ear [6,8,9]. Interestingly, inner ear NER factors can demonstrate nuclear translocation to toxic chemical and physical exposures, and it is believed that NER is a general protective response in the mammalian inner ear [39]. More recent experiments have shown that noise exposure or telomere treatment were both effective at inducing inner ear expression of NER factors [7]. Yet, telomere’s induction of NER resulted in the preservation of inner ear functions while noise induction of NER resulted in disordered inner ear functions [7]. The underlying basis for this discrepancy is unknown and may involve multiple mechanisms. In the current series of experiments, it was discovered that noise stress failed to induce the expression of *Xpg*, a structure-specific endonuclease that is critical to NER. This finding was further confirmed when noise stress also failed to induce the expression of *Xpf*, another vital structure-specific endonuclease. The biological significance of noise-induced abrogation of *Xpg*, and *Xpf* was explored in experiments focused on general inner ear functions, particularly nonlinear signal processing via distortion product levels. These experiments revealed that the noise-induced abrogation of *Xpg* and *Xpf* was associated with disordered nonlinear signal processing. This suggests that noise-induced mobilization of inner ear NER might be corrupted due to abrogated expression of structure-specific endonucleases. Given that noise stress is known to induce DNA damage within the inner ear, the current series of experiments are suggestive of a role for structure-specific endonucleases in the pathobiology of noise-induced inner ear dysfunctions.

NER is a versatile biochemical process that detects and excises a large variety of structurally and chemically distinct DNA damage products [1,2]. The NER process involves multiple steps, including the assembly of a pre-incision protein complex, excision of oligonucleotides around the site of damage, post-excision degradation of the excised/damaged oligonucleotide, and finally, the synthesis of a new oligonucleotide to replace the old/damaged oligonucleotide. The XPG and XPF endonucleases are important to every step [10,11,12]. In the assembly of the pre-incision protein complex, XPG plays a structural role by stabilizing the entire complex via its interaction with TFIIH subunits. Here the N-terminal and C-terminal regions of XPG are bound to TFIIH subunits, and this interaction is necessary for the NER process to continue [12]. Furthermore, XPF interacts with XPA via the XPA binding motif within ERCC1, and this complex interaction is necessary for a functional pre-incision protein complex [10]. Therefore, conditions that abrogate the expression of *Xpg* and/or *Xpf* would be expected to reduce the efficiency of the pre-incision step. In the current experiments, noise stress abrogated the expression of both *Xpg* and *Xpf* within the mammalian inner ear, and this effect would be expected to reduce the efficiency of inner ear NER.

After the assembly of the pre-incision protein complex around a given DNA lesion, the next step in the NER process is excision of oligonucleotides around the site of damage. This step is solely the responsibility of XPF and XPG [12]. First, the nuclease domain within XPF makes a 5′-incision ~20 ± 5 phosphodiester bonds upstream of the damaged DNA [40,41]. This creates a free 3′-OH tail that serves as a substrate for initiating polymerization by pol*δ*, polε and polκ [42]. After polymerization has begun, XPG induces a 3′-incision that is 6 ± 3 phosphodiester bonds downstream of the damaged DNA, which effectively excises a 24–32 stretch of oligonucleotide that contains the damaged DNA [40,41,43]. After this excision, the post-excision degradation of the excised/damaged oligonucleotide can commence. Even at this late stage, both XPG and XPF play important roles. For instance, both XPG and XPF stay bound to the damaged oligonucleotide to ensure binding to RPA prior to nucleolytic degradation of the entire oligonucleotide [11,44,45]. The final NER step is the re-synthesis of a new strand of DNA. Here, the 5′-incison made by XPF is critical for initiating DNA polymerization, while the PCNA interaction motif within XPG has been shown to further facilitate the re-synthesis step [11,19,46,47].

Dual incision by XPF and XPG is the hallmark step within the NER process [11]. However, both XPF and XPG assume important roles within other NER steps, and conditions that abrogate the expression of *Xpf* and *Xpg* will effectively eliminate NER [12]. In contrast, conditions that potentiate the expression of *Xpf* and *Xpg* could enhance the efficiency of NER. In the current experiments, the delivery of telomere into the inner ear resulted in an increase in the expression of *Xpf* and *Xpg*. Further experiments showed that as the concentration of telomere increased, there was a corresponding increase in the expression of both *Xpf* and *Xpg*. Additionally, this telomere-induced expression was stable for at least 3 days after induction. Interestingly, these molecular-level effects were associated with improved inner ear functions after severe noise stress. Given that noise stress causes DNA damage within inner ear cells and DNA damage will disrupt the function of inner ear genes, it is tempting to speculate that the telomere-induced expression of *Xpf* and *Xpg* enhanced the efficiency of NER, which protected the genome of cells within the inner ear, leading to improved inner ear functions. However, such machinations need to be followed with additional mechanistic experiments. For instance, it is still not clear why noise exposure would augment the expression of multiple NER genes but abrogate the expression of the two structure-specific endonucleases. Nonetheless, the current results provide a basis for pursuing such experiments in the future. Additionally, both *Xpg* and *Xpf* were downregulated with noise and upregulated with telomere—an indication they might be co-regulated in the mammalian inner ear. This is consistent with the fact they complement each other in NER. However, it is not known whether one of these endonucleases (XPG or XPF) is more protective than the other. Further, it is not known why cochlear expression of *Xpg* is more abundant than that of *Xpf* under control and experimental conditions. Therefore, the current experiments have provided a basis for conducting future studies that employ conditional knockdown of each endonuclease.

The current work is significant because it revealed that noise exposure abrogates the expression of *Xpg* and *Xpf*. Research on noise exposure and NER has demonstrated that noise exposure increases the transcriptional expression of NER genes above normal (control) conditions [7]. Given that NER is a protective mechanism, this noise-induced increase in NER genes would be expected to provide some level of protection. Instead, a noise-induced increase in NER genes above normal/control conditions is consistently associated with the loss of cochlear functions, such as nonlinear signal processing. This mystery has remained unsolved; however, the current work suggest that noise exposure may limit the transcriptional increase in *Xpg* and *Xpf* (prevent an increase above normal/control conditions), two compulsory NER factors. Interestingly, both *Xpg* and *Xpf* are in fact inducible. The current work showed that both *Xpg* and *Xpf* can be induced when noise + telomere is introduced and when telomere alone is introduced. However, noise alone failed to induce endonuclease expression. Combined, the current evidence suggests that noise exposure abrogates the expression of *Xpg* and *Xpf*, which may explain why noise-induced NER is pathological while therapeutic (e.g., telomere) induction of NER is protective.

## 4. Materials and Methods

### 4.1. Research Design

Experimentally naïve male Long–Evans rats (1 month old) served as subjects during the experiments. Preliminary studies did not reveal differences between male and female rats; therefore, males were used in the current experiments. All rats were purchased from Harlan Laboratories, Inc. (Livermore, CA, USA). The animals were maintained in an Association for Assessments and Accreditation of Laboratory Animal Care (AAALAC)-accredited vivarium, where they had free access to food and water. All animal protocols received Institutional Animal Care and Use Committee approval. Animal protocols were in compliance with the following regulations: (1) Title 9 Code of Federal Regulations, Chapter 1, Subchapter A: Animal Welfare; (2) Department of Defense Instruction 3216.01; (3) Air Force Manual (AFMAN) 40-401; and (4) the Guide for the Care and Use of Laboratory Animals, Institute of Laboratory Animal Resources, National Research Council. The animal protocols were designed to reduce the number of animals sacrificed for the experiments and minimize animal pain and discomfort. Animals were randomly selected into the following four groups: control, noise, telomere, and noise + telomere. Normal cochlear receptor function (DPOAE recordings) was verified among all animals prior to any manipulations (i.e., baseline). The control group started and ended the study with the experimental groups, but this group did not receive noise exposure or telomere treatment. Except for noise exposure and telomere treatment, the control group received the same experiences (e.g., anesthesia, handling and husbandry) as the experimental groups. The noise group was exposed to noise; then, 24 h later, the inner ears from this group were dissected, and their cochleae flash-frozen. The telomere group was treated with telomere (no noise exposure), which was dissolved in saline (vehicle); then, 24 h later, the inner ears from this group were dissected and flash-frozen. Pilot experiments showed that the delivery of saline-vehicle to the inner ear had no effect on gene expression. The animals in the noise + telomere group were first exposed to noise such that both the right and left inner ears had equal probability of being exposed to the noise. When the noise exposure ended, one inner ear immediately received telomere, while the opposite ear received saline-vehicle. Then, 24 h later, both the right and left inner ears from each animal were dissected, and their cochleae flash-frozen. A subgroup of animals from the noise + telomere group were allowed to survive for functional assessments. The function of receptor cells within both cochleae from this group was evaluated at 7 and 60 days after the noise exposure. For gene expression studies, there were three biological replicates per group (*n* = 3/group). In the dose–response and time-course studies, a total of seven animals were used (*n* = 7). Lastly, five biological replicates (*n* = 5) were used in the functional studies.

### 4.2. Noise Stress

The noise exposure apparatus has been described in detail previously [9]. Briefly, the animals in the noise-only and noise + telomere groups were exposed to an 8 kHz OBN @ 105 dB SPL for 16 h. This noise stress is known to kill receptor cells in the inner ear and induce permanent inner ear dysfunction in Long–Evans rats. The animals were awake for the noise exposure and were placed in 15 × 13 × 11 cm wire-cloth enclosures within a cylindrical 40 L reverberant chamber. A DS335 Function Generator (Stanford Research Systems, Menlo Park, CA, USA) was used to generate broadband noise, while a Frequency Device 9002-Dual-Channel Filter/Amplifier Instrument (Frequency Device Inc., Haverhill, MA, USA) with a roll-off of 48 dB/octave morphed the broadband noise into an OBN with center frequency at 8 kHz. An HCA1000A Parasound Amplifier (Parasound Products, Inc., San Francisco, CA, USA) was then used to amplify and deliver the OBN to Vifa D25AG-05 speakers (Vifa International A/S, Videbaek, Denmark) that were located approximately 5 cm above the animals’ wire-cloth enclosure. Sound pressure levels measured at the rats’ ears were 105 dB SPL in the octave band centered around 8 kHz.

### 4.3. Telomere Delivery

The chemical synthesis and delivery of telomere into the ear have been described in detail previously [7]. Briefly, telomere (also called t-oligo) is a synthetic oligonucleotide with 100% sequence homology to the single-strand 3′ tandem repeats (5′-TTAGGG-3′) of the mammalian telomere overhang. It was prepared using cyanoethyl phosphoramidite chemistry (Midland Certified Regent Company, Midland, TX, USA) and diluted in sterile physiological saline at concentrations ranging from 1 to 10.8 mM. Telomere was delivered directly to the ear via transtympanic injections (TTIs). Animals in the telomere and noise + telomere groups received a single TTI of telomere. First, the animals received ketamine/xylazine (44/7 mg/kg, i.m.) anesthesia, and their blink-to-threat reflex was monitored to ensure appropriate levels of anesthesia depth. Each animal’s tympanic membrane was then otoscopically evaluated and found to be free of debris, translucent, and concave, exhibiting a positive cone-of-light and visible short process of the malleus. Both the *pars flaccida* and *pars tensa* were visible. Telomere (dissolved in saline) was administered through a single myringotomy made within the tympanic membrane. For dose–response studies, the rats received telomere at concentrations that ranged from 1 to 10.8 mM. A concentration of 3.6 mM was the lowest most effective dose; therefore, this concentration (dose) was used in all other experiments.

### 4.4. Real-Time Polymerase Chain Reactions

#### 4.4.1. Tissue Procurement

Under general anesthesia (ketamine/xylazine 44/7 mg/kg, i.m.) and after disappearance of the blink-to-threat and the paw-withdrawal reflexes, each animal was decapitated. The scalp was then removed, followed by a gradual peeling of the cartilaginous portion of the external auditory meatus to expose the osseous bulla. The bulla was carefully resected to expose the cochlea, which was then dissected from the temporal bone. Dissected specimens were then flash-frozen in liquid nitrogen. All experimental conclusions were based on pooled data from triplicate biological replicates [48].

#### 4.4.2. Spin Column Chromatography

Each frozen specimen was pulverized in an SKP buffer (Norgen BioTEK corp., Thorold, ON, Canada) to create a lysate solution. DNA was then captured and eliminated via a genomic DNA removal column (Norgen BioTEK corp., Thorold, ON, Canada). Ethanol was infused to precipitate nucleic acids within the DNA-free solution. A proprietary nucleic acid resin (Norgen corp.) was used to bind and stabilize mRNA, rRNA and miRNA; then, impurities and insoluble products were removed. The centrifugation protocol was as follows: 2 min at 14,000× *g* to isolate and remove pellet from lysis solution; 1 min at 5200× *g* to isolate and remove genomic DNA; 1 min at 14,000× *g* for RNA binding to column resin; 3 × 1 min at 3500× *g* to remove impurities from RNA; 2 min at 14,000× *g* to dry the resin; 2 min at 200× *g*, then 1 min at 14,000× *g* to elute RNA from the resin. Nanodrop 2000 (Thermo Scientific, Waltham, MA, USA) was used to determine RNA concentrations. Purified total RNA was stored at −80 °C for later use.

#### 4.4.3. First-Strand cDNA Synthesis

Poly(A) + mRNA was converted to cDNA via reverse transcription. Total RNA (1 μg) was incubated at 70 °C for 5 min, spun on a microcentrifuge, then cooled on ice for 5 min. A 25 μL reverse transcription reaction was initiated with the following reactants: 0.5 μg Oligo(dT)_15_ (Promega, Madison, WI, USA); 1 μg total RNA; 5 μL of M-MLV RT 5× reaction buffer, 4 μL of dNTP mix (10 mM each), 100 Units of M-MLV RT (H-) point mutant reverse transcriptase (Promega, USA), and nuclease-free H_2_O. Following agitation, the reaction proceeded with incubation at 40 °C for 10 min, then 42 °C for 50 min. The reaction was then inactivated by heating for 15 min at 70 °C.

#### 4.4.4. Real-Time Fluorescence Polymerase Reaction

The reaction consisted of: 10 μL of 2× SYBR green PCR master mix (Applied Biosystems, Inc., Waltham, MA, USA), 1 μL of gene primer mix (10 μM each), 5 μL of cDNA (4× dilution from first-strand cDNA) in a final volume of 20 μL. The thermo-cycling protocol was as follows: 1 min at 95 °C, 40 cycles at 95 °C for 20 s and 30 s at 60 °C. The following rat primers were used: *Xpf*-specific primers (forward 5′-CAGCCTCAACTCATGCATCTA-3′ and reverse, 5′ CGACCTCACACTCACTACTTATC-3) and *Xpg*-specific primers (forward, 5′ CAGCCTCAACTCATGCATCTA-3′ and reverse, 5′-TCTCTTTCAGGGCATGGTTGG-3′). The 18S ribosomal ribonucleic acid (rRNA) was used as a housekeeping gene (forward, 5′-ACCGGCGCAAGACGAACCAG-3′ and reverse, 5′-GCATCGCCAGTCGGCATCGT-3′).

#### 4.4.5. Data Analysis

The 6-carboxy-X-rhodamine (6-ROX) fluorophore was used as an internal reference in the real-time fluorescent polymerase reactions. 6-ROX does not participate in amplicon production and thus served to normalize the fluorescent signal generated by SYBR-green intercalated within dsDNA. Baseline fluorescence generated by 6-ROX was subtracted from SYBR-dsDNA fluorescence as the polymerase reaction progressed over time. This generated real-time changes in the emission spectra (Δ*R_n_*). The Δ*R_n_* as a function of polymerization was plotted for each gene and each experimental condition. The polymerization cycle at which the SYBR-dsDNA fluorescence crossed the midlinear slope of the Δ*R_n_*–polymerization plot was denoted as C_T_. To quantify gene expression level, the C_T_ was converted to 2^− ΔC^_T_, where ∆C_T_ = C_T(Target Gene)_ − C_T(18S rRNA)_ [49]. The Step-One Plus^TM^ Real-time PCR Software v2.0 (Applied Biosystems Inc., Waltham, MA, USA) was used for instrument control, automated data collection and data analysis. The 2 ^− ΔC^_T_ data were treated with separate one-way analysis of variance (ANOVA), where condition (control, noise, telomere and noise + telomere) served as the between-group factor for the expression of either *Xpf* or *Xpg* [6]. Dunnett’s pairwise comparisons were then used to determine statistically significant differences between groups. A *p*-value < 0.05 was accepted as statistically significant.

### 4.5. Inner Ear Function

#### 4.5.1. Animals

Permanent changes in nonlinear signal processing were evaluated by recording DPOAEs at baseline as well as 7 and 60 days after the noise stress. Each animal was anesthetized with ketamine (44 mg/kg) and xylazine (7 mg/kg), while body temperature was maintained at 37 °C with a DC-heating unit build into a 7″ × 15″ surgical table. A double-walled anechoic chamber (Industrial Acoustics Company Inc., Bronx, NY, USA) was used for all recordings, and the animals were staged in a lateral position with the test pinna directly facing the probe. These recordings lasted 10 min per animal. All experimental conclusions were based on pooled data from five biological replicates.

#### 4.5.2. Apparatus

A probe assembly was hermetically sealed to the external auditory meatus of each animal via an ER3-34 silicon tip (Etymotic Research, Elk Grove Village, IL, USA). The seal was strong enough that the head of the animal could be raised off the surgical table by elevating the probe assembly. The probe assembly consisted of two polyethylene tubes coupled to two separate Realistic dual radial horn tweeters (Radio Shack, Tandy Corp., Fort Worth, TX, USA). These tweeters were used to present stimulating pure-tone primaries: *f_1_* and *f_2_*. The probe assembly also consisted of a pre-amplifier microphone cable coupled to an ER-10B+ emission microphone (Etymotic Research). All elements of the probe assembly were controlled through a customized signal presentation, acquisition, and analysis algorithm written in LabVIEW version 7.1 (National Instruments, Austin, TX, USA). This LabVIEW algorithm was also used to drive a PCI-4461 computer-based digital signal processing board (National Instruments). The signal processing algorithm, employed in the acquisition and analysis of DPOAE responses, and its validation have been reported previously [50,51].

#### 4.5.3. Electroacoustics and Biogenic Responses

The nonlinear signal processing of receptor hair cells was evaluated with two pure-tone primaries *f*_2_ and *f*_1_, where *f*_2_ was higher in frequency than *f*_1_ (*f*_2_/*f*_1_ frequency ratio of 1.25). Recordings were standardized across ears and across animals. Here, 75 dB SPL pure tones at 1 and 2 kHz were presented to each ear, while in situ meatal recordings ensured that the pure tones were the expected frequency and the expected level. If the tones did not meet the desired frequency and level, the probe assembly was repositioned, and the procedure was repeated until the standard was met. This procedure ensured the reliability of every recording. The noise floor (instrumental and biologic) was computed by averaging SPLs from the external auditory meatus for frequency bins above and below each distortion product bin (50–100 Hz). A 0.2-cm^3^ hard-walled coupler that approximated the rat’s external auditory meatus was used to monitor the quality of the recordings, identify nonbiogenic responses, and check the level of the instrumental noise floor. These quality measurements were free of artifacts and did not produce distortions that exceeded the noise floor.

#### 4.5.4. Data Analysis

Planned pairwise comparisons were made between noise and noise + telomere conditions at Seven and 60 days post-noise exposure. Therefore, Bonferroni statistical computations were conducted on DPOAE levels to determine significant differences between noise and noise + telomere conditions. *p*-values < 0.05 were considered statistically significant for all computations.

## 5. Conclusions

NER is a multistep biochemical process that maintains the integrity of the genome. Unlike other mechanisms that maintain genomic integrity, NER is distinguished by two irreversible nucleolytic events that are executed by the XPG and XPF structure-specific endonucleases. Beyond nucleolysis, XPG and XPF regulate the overall efficiency of NER through various protein–protein interactions. Therefore, environmental conditions that negatively affect the expression of *Xpg* and/or *Xpf* would increase genomic instability to ultimately perpetuate pathophysiologic outcomes. The current experiments revealed that exposure to noise abrogated the expression of both *Xpg* and *Xpf*, and these effects were associated with pathological nonlinear signal processing from receptor cells within the mammalian inner ear. Given that exposure to environmental sounds (noise, music, etc.) is ubiquitous in daily life, sound-induced corruption of NER might represent an overlooked genomic threat.

## Figures and Tables

**Table 1:** Statistical computations on endonuclease expression levels.

Source	df	SS	MS	F-Values
***Xpg***:				
Between conditions	3	57.94	19.31	10.65 ^a^
Within/Residual	8	14.51	1.814	
Total	11	72.45		
***Xpf*: **				
Between conditions	3	0.008	0.025	0.3901
Within/Residual	8	0.018	0.0022	
Total	11	0.025		

Note: Degrees of freedom (*df*), sums of squares (*SS*), mean squares (*MS*) and F-values are shown for separate one-way ANOVAs, where condition (control, noise, telomere and noise+telomere) served as the between-group factor for the expression of either *Xpf* or *Xpg*. Main effects of condition are indicated. ^a^ *p* < 0.01.

## Data Availability

Data are available from the author upon reasonable request.

## References

[1] Brooks P.J. (2017). The Cyclopurine Deoxynucleosides: DNA Repair, Biological Effects, Mechanistic Insights, and Unanswered Questions. Free Radic. Biol. Med..

[2] Gillet L.C.J., Schärer O.D. (2006). Molecular Mechanisms of Mammalian Global Genome Nucleotide Excision Repair. Chem. Rev..

[3] Viana L.M., Seyyedi M., Brewer C.C., Zalewski C., DiGiovanna J.J., Tamura D., Totonchy M., Kraemer K.H., Nadol J.B. (2013). Histopathology of the Inner Ear in Patients with Xeroderma Pigmentosum and Neurological Degeneration. Otol. Neurotol..

[4] Guthrie O.W. (2008). Preincision Complex-I from the Excision Nuclease Reaction among Cochlear Spiral Limbus and Outer Hair Cells. J. Mol. Histol..

[5] Guthrie O.W. (2015). Localization and Distribution of Neurons That Co-Express Xeroderma Pigmentosum-A and Epidermal Growth Factor Receptor within Rosenthal’s Canal. Acta Histochem..

[6] Guthrie O.W., Carrero-Martínez F.A. (2010). Real-Time Quantification of Xeroderma Pigmentosum mRNA from the Mammalian Cochlea. Ear Hear..

[7] Guthrie O.W. (2017). Functional Consequences of Inducible Genetic Elements from the P53 SOS Response in a Mammalian Organ System. Exp. Cell Res..

[8] Guthrie O.W., Li-Korotky H.-S., Durrant J.D., Balaban C. (2008). Cisplatin Induces Cytoplasmic to Nuclear Translocation of Nucleotide Excision Repair Factors among Spiral Ganglion Neurons. Hear. Res..

[9] Guthrie O.W., Xu H. (2012). Noise Exposure Potentiates the Subcellular Distribution of Nucleotide Excision Repair Proteins within Spiral Ganglion Neurons. Hear. Res..

[10] Faridounnia M., Folkers G.E., Boelens R. (2018). Function and Interactions of ERCC1-XPF in DNA Damage Response. Molecules.

[11] Kemp M.G., Hu J. (2017). PostExcision Events in Human Nucleotide Excision Repair. Photochem. Photobiol..

[12] Fagbemi A.F., Orelli B., Schärer O.D. (2011). Regulation of Endonuclease Activity in Human Nucleotide Excision Repair. DNA Repair..

[13] Constantinou A., Gunz D., Evans E., Lalle P., Bates P.A., Wood R.D., Clarkson S.G. (1999). Conserved Residues of Human XPG Protein Important for Nuclease Activity and Function in Nucleotide Excision Repair. J. Biol. Chem..

[14] Wakasugi M., Reardon J.T., Sancar A. (1997). The Non-Catalytic Function of XPG Protein during Dual Incision in Human Nucleotide Excision Repair. J. Biol. Chem..

[15] Scherly D., Nouspikel T., Corlet J., Ucla C., Bairoch A., Clarkson S.G. (1993). Complementation of the DNA Repair Defect in Xeroderma Pigmentosum Group G Cells by a Human cDNA Related to Yeast RAD2. Nature.

[16] Dunand-Sauthier I., Hohl M., Thorel F., Jaquier-Gubler P., Clarkson S.G., Schärer O.D. (2005). The Spacer Region of XPG Mediates Recruitment to Nucleotide Excision Repair Complexes and Determines Substrate Specificity. J. Biol. Chem..

[17] He Z., Henricksen L.A., Wold M.S., Ingles C.J. (1995). RPA Involvement in the Damage-Recognition and Incision Steps of Nucleotide Excision Repair. Nature.

[18] Iyer N., Reagan M.S., Wu K.-J., Canagarajah B., Friedberg E.C. (1996). Interactions Involving the Human RNA Polymerase II Transcription/Nucleotide Excision Repair Complex TFIIH, the Nucleotide Excision Repair Protein XPG, and Cockayne Syndrome Group B (CSB) Protein. Biochemistry.

[19] Gary R., Ludwig D.L., Cornelius H.L., MacInnes M.A., Park M.S. (1997). The DNA Repair Endonuclease XPG Binds to Proliferating Cell Nuclear Antigen (PCNA) and Shares Sequence Elements with the PCNA-Binding Regions of FEN-1 and Cyclin-Dependent Kinase Inhibitor P21. J. Biol. Chem..

[20] Warbrick E. (1998). PCNA Binding through a Conserved Motif. BioEssays.

[21] Evans E., Moggs J.G., Hwang J.R., Egly J.-M., Wood R.D. (1997). Mechanism of Open Complex and Dual Incision Formation by Human Nucleotide Excision Repair Factors. EMBO J..

[22] Choi Y.-J., Ryu K.-S., Ko Y.-M., Chae Y.-K., Pelton J.G., Wemmer D.E., Choi B.-S. (2005). Biophysical Characterization of the Interaction Domains and Mapping of the Contact Residues in the XPF-ERCC1 Complex. J. Biol. Chem..

[23] de Laat W.L., Sijbers A.M., Odijk H., Jaspers N.G.J., Hoeijmakers J.H.J. (1998). Mapping of Interaction Domains between Human Repair Proteins ERCC1 and XPF. Nucleic Acids Res..

[24] Gaillard P.-H.L., Wood R.D. (2001). Activity of Individual ERCC1 and XPF Subunits in DNA Nucleotide Excision Repair. Nucleic Acids Res..

[25] Tripsianes K., Folkers G., Ab E., Das D., Odijk H., Jaspers N.G.J., Hoeijmakers J.H.J., Kaptein R., Boelens R. (2005). The Structure of the Human ERCC1/XPF Interaction Domains Reveals a Complementary Role for the Two Proteins in Nucleotide Excision Repair. Structure.

[26] Fisher L.A., Bessho M., Wakasugi M., Matsunaga T., Bessho T. (2011). Role of Interaction of XPF with RPA in Nucleotide Excision Repair. J. Mol. Biol..

[27] Kopke R.D., Jackson R.L., Coleman J.K.M., Liu J., Bielefeld E.C., Balough B.J. (2007). NAC for Noise: From the Bench Top to the Clinic. Hear. Res..

[28] Nelson D.I., Nelson R.Y., Concha-Barrientos M., Fingerhut M. (2005). The Global Burden of Occupational Noise-Induced Hearing Loss. Am. J. Ind. Med..

[29] Zelaya C., Lucas J., Hoffman H. (2015). Self-Reported Hearing Trouble in Adults Aged 18 and Over: United States, 2014. NCHS Data Brief.

[30] Clark W.W. (1991). Noise Exposure from Leisure Activities: A Review. J. Acoust. Soc. Am..

[31] Muchnik C., Amir N., Shabtai E., Kaplan-Neeman R. (2012). Preferred Listening Levels of Personal Listening Devices in Young Teenagers: Self Reports and Physical Measurements. Int. J. Audiol..

[32] Guthrie O.W. (2017). Noise Induced DNA Damage Within the Auditory Nerve. Anat. Rec..

[33] Van Campen L.E., Murphy W.J., Franks J.R., Mathias P.I., Toraason M.A. (2002). Oxidative DNA Damage Is Associated with Intense Noise Exposure in the Rat. Hear. Res..

[34] Kamio T., Watanabe K.-I., Okubo K. (2012). Acoustic Stimulation Promotes DNA Fragmentation in the Guinea Pig Cochlea. J. Nippon. Med. Sch..

[35] Bialek W., Wit H.P. (1984). Quantum Limits to Oscillator Stability: Theory and Experiments on Acoustic Emissions from the Human Ear. Phys. Lett. A.

[36] Robles L., Ruggero M.A. (2001). Mechanics of the Mammalian Cochlea. Physiol. Rev..

[37] Kemp D.T. (2002). Otoacoustic Emissions, Their Origin in Cochlear Function, and Use. Br. Med. Bull..

[38] Avan P., Büki B., Petit C. (2013). Auditory Distortions: Origins and Functions. Physiol. Rev..

[39] Guthrie O.W. (2012). Dynamic Compartmentalization of DNA Repair Proteins within Spiral Ganglion Neurons in Response to Noise Stress. Int. J. Neurosci..

[40] Huang J.C., Svoboda D.L., Reardon J.T., Sancar A. (1992). Human Nucleotide Excision Nuclease Removes Thymine Dimers from DNA by Incising the 22nd Phosphodiester Bond 5′ and the 6th Phosphodiester Bond 3′ to the Photodimer. Proc. Natl. Acad. Sci. USA.

[41] Staresincic L., Fagbemi A.F., Enzlin J.H., Gourdin A.M., Wijgers N., Dunand-Sauthier I., Giglia-Mari G., Clarkson S.G., Vermeulen W., Schärer O.D. (2009). Coordination of Dual Incision and Repair Synthesis in Human Nucleotide Excision Repair. EMBO J..

[42] Ogi T., Limsirichaikul S., Overmeer R.M., Volker M., Takenaka K., Cloney R., Nakazawa Y., Niimi A., Miki Y., Jaspers N.G. (2010). Three DNA Polymerases, Recruited by Different Mechanisms, Carry out NER Repair Synthesis in Human Cells. Mol. Cell.

[43] Edenberg H., Hanawalt P. (1972). Size of Repair Patches in the DNA of Ultraviolet-Irradiated HeLa Cells. Biochim. Biophys. Acta (BBA) Nucleic Acids Protein Synth..

[44] Hu J., Choi J.-H., Gaddameedhi S., Kemp M.G., Reardon J.T., Sancar A. (2013). Nucleotide Excision Repair in Human Cells: Fate of the Excised Oligonucleotide Carrying DNA Damage In Vivo. J. Biol. Chem..

[45] Kemp M.G., Reardon J.T., Lindsey-Boltz L.A., Sancar A. (2012). Mechanism of Release and Fate of Excised Oligonucleotides during Nucleotide Excision Repair. J. Biol. Chem..

[46] Han C., Wani G., Zhao R., Qian J., Sharma N., He J., Zhu Q., Wang Q.-E., Wani A.A. (2015). Cdt2-Mediated XPG Degradation Promotes Gap-Filling DNA Synthesis in Nucleotide Excision Repair. Cell Cycle.

[47] Mocquet V., Lainé J.P., Riedl T., Yajin Z., Lee M.Y., Egly J.M. (2008). Sequential Recruitment of the Repair Factors during NER: The Role of XPG in Initiating the Resynthesis Step. EMBO J..

[48] Kitahara T., Li-Korotky H.S., Balaban C.D. (2005). Regulation of Mitochondrial Uncoupling Proteins in Mouse Inner Ear Ganglion Cells in Response to Systemic Kanamycin Challenge. Neuroscience.

[49] Schmittgen T.D., Livak K.J. (2008). Analyzing Real-Time PCR Data by the Comparative CT Method. Nat. Protoc..

[50] Martin G.K., Stagner B.B., Chung Y.S., Lonsbury-Martin B.L. (2011). Characterizing Distortion-Product Otoacoustic Emission Components across Four Species. J. Acoust. Soc. Am..

[51] Martin G.K., Stagner B.B., Lonsbury-Martin B.L. (2006). Assessment of Cochlear Function in Mice: Distortion-Product Otoacoustic Emissions. Curr. Protoc. Neurosci..

